# The Mereotopology of Pregnancy

**DOI:** 10.1093/jmp/jhad017

**Published:** 2023-04-15

**Authors:** Suki Finn

**Affiliations:** Royal Holloway University of London, London, United Kingdom

**Keywords:** *maximality*, *mereology*, *metaphysics*, *pregnancy*, *topology*

## Abstract

Consider the following two metaphysical questions about pregnancy: (1) When does a new organism of a certain kind start to exist? (2) What is the mereological and topological relationship between the pregnant organism and with what it is pregnant? Despite assumptions made in the literature, I take these questions to be independent of each other, such that an answer to one does not provide an answer to the other. I argue that the way to connect them is via a maximality principle that prevents one organism being a proper part of another organism of the same kind. That being said, such a maximality principle need not be held, and may not apply in the case of pregnancy. The aims of this paper are thus to distinguish and connect these metaphysical questions, in order to outline a taxonomy of rival mereotopological models of pregnancy that result from the various combinations of their answers.

## I. CLARIFYING THE DEBATE

Recently, and most prominently in the *Journal of Medicine and Philosophy*, a new debate has arisen over metaphysical aspects of pregnancy. For example, Barry Smith and Berit Brogaard argue that a new organism starts to exist at sixteen days, whereas, using the same criterion, Elselijn Kingma responds that a new organism would start to exist at birth.^1^ What is new about this debate is that it makes explicit reference to the metaphysical relationship between the pregnant organism and with what it is pregnant, and it is this connection (between *when a new organism starts to exist* and *the maternal-fetal relationship*) that I wish to challenge. The metaphysics of pregnancy fits into the domain that Katherine Hawley calls “applied metaphysics.” According to her construal, applied metaphysics concerns “metaphysical issues which lend themselves to application beyond philosophy [and] are often (though not always) issues to do with categorising, classifying and organising the world” (Hawley, 2016, 1). Pregnancy is clearly a topic that applies outside of philosophy: many of us have been, will be, or are pregnant, and all of us are the result of a pregnancy. Now, this paper shows how a philosophical inquiry of pregnancy helps to categorize, classify, and organize the entities that are involved in a pregnancy.

Given the currently minimal amount of relevant literature, the main aspect of this paper is to clarify the questions and their possible answers, rather than arguing for one of the particular answers. The project of adjudicating among the possibilities I outlined in order to answer is actually the question I embark on here, but I do hope that I help lay the groundwork for it here. So, in the remainder of Section I, I spend time defining terminology. In Section II, I outline what I call the “Relationship Question” regarding the metaphysical relationship between the pregnant organism and that with which it is pregnant, and distinguish this from what I call the “Timing Question” regarding when a new organism starts to exist. After providing possible answers to these questions, I combine them with each other to provide some form of taxonomy for available positions in the debate. Finally, I draw together the lessons learnt from this metaphysical exploration of pregnancy.

First, let us examine terminology. The thing that is pregnant I call the “Gravida,” and the thing the Gravida is pregnant with I call the “Foster.” Following Smith and Brogaard and Kingma, “Foster” is used to generally pick out the entity with which the Gravida is pregnant at any stage of pregnancy, such as the zygote, embryo, or fetus.^2^ Kingma uses “Gravida” to refer to the pregnant organism regardless of the type of organism it is (so long as it is a placental mammal) and regardless of their social status as a “mother.” Smith and Brogaard, like many others in the *Journal of Medicine and Philosophy*, use the term “human being” as synonymous with “human organism.”^3^ Given that generally, organisms give birth to organisms of the same kind, our Timing Question specifically asks when an organism of the same kind as the (pre-pregnant) Gravida starts to exist. In this paper, I refer to this sameness of kind as “relevant kind.”^4^ Our Relationship Question and Timing Question can be articulated for now as such:

*Timing Question*: When does an organism of the relevant kind start to exist?

*Relationship Question*: Is the Foster a part of, or contained by, the Gravida?^5^

Given that there is meant to be a *choice* in the Relationship Question, we must be able to distinguish parthood from containment as contrast classes, which is difficult when parthood is a mereological notion and containment a topological notion. If we want to allow for the possibilities of uncontained parts and contained nonparts, our understanding of containment must not entail or be entailed by parthood. Contained nonparts are easy to imagine. One example is an oven with buns cooking inside it—the buns are not part of the oven, yet the oven contains them. Contained parts are also easy to imagine, for example, a shelf in the oven that the buns are cooking on—the shelf is not *only* contained by the oven, but is also part of the oven. The difficult case to imagine is uncontained parts. This is because if a whole just *is* where its parts are, then all parts are in some way contained by the whole, as the whole will encapsulate all the parts. Now this does not capture the sense of containment in which we are interested, which is more to do with being *inside*. So, if we take the shelves of the oven out of the oven to clean them, we say we are cleaning part of the oven (as the shelves are parts of the oven) but they are not inside the oven, and thus are uncontained parts.^6^

Note, that we are only interested in *proper* parthood, where the part does not equal the whole.^7^ So, the shelves are a proper part of the oven, whereas the oven itself is an improper part of the oven. We are not questioning whether the Foster *is* the Gravida—rather, we are questioning whether there is a proper part of the Gravida that we identify as the Foster. As such, from hereon, wherever I speak of “part” I mean “proper part,” unless otherwise stated by talking of an “improper part.” Also, in order to forge a contrast class between this proper parthood and containment, I define and distinguish the variants of containment as such:

*Containment*: x contains y iff y is within the external bona fide boundary of x.^8^

*Part-containment*: x part-contains y iff y is contained by x, and y is a proper part of x.

*Mere-containment*: x merely-contains y iff y is contained by x, and y is not a part of x.^9^

Kaiser describes external boundaries as being of “particular importance to the identity of many biological objects” (2018, 73). For example, Kaiser notes that the natural external boundary of a mammal would be its skin, because it is the skin that helps us to identify and individuate the mammal. As such, a mammal contains anything within the boundary of their skin, including their organs (part-containment) and the food being digested (mere-containment). Now notice that this distinction does not carve the interesting joints at all. For example, there seem to be two different ways of being part-contained: trivially and nontrivially. Take one of my organs, and subtract or add one of my cells from it or to it. The organ ± cell is part-contained within me because it still qualifies as being part of me and within my external boundary. Any arbitrary demarcation of me results in something that is part-contained within me. It would be helpful to differentiate this rather trivial sense of part-containment from the nontrivial part-containment relationship between me and my organ. To do so would require a notion of individuation that would capture the distinguished parts that I contain from the trivial parts that I contain. I suggest making this distinction again with the help of the notion of bona fide boundaries. There is a bona fide boundary between my organ (as a nontrivial part) and the remainder of me, and there is a fiat boundary between arbitrary demarcations of me (like my organ ± cell, as a trivial part).

My distinction between part-containment and mere-containment has the following results: no proper parts of a whole are *merely* contained by the whole; some proper parts of a whole are contained by the whole (as this is *part*-containment), and not all contained things are proper parts of the container (as this is *mere*-containment). It seems plausible that the Gravida *generally* contains the Foster, because during pregnancy the Foster is located within the external boundary of the Gravida. So, given that we can take it as common ground between all parties that the Foster is contained by the Gravida, the important question that divides people is whether that contained Foster is *merely* contained, or *part* contained, that is, whether the Foster is a proper part of the Gravida or not. Thus, the interesting issue lies in the choice between *proper part* and *mere-containment*, or—in other words—between parthood and non-parthood. With these distinctions in mind, we can further refine the Relationship Question to be as such:

*Relationship Question*: Is the Foster a proper part of, or merely contained by, the Gravida?

As I demonstrate, in the current literature, an answer to the Timing Question is seen as depending in part on the answer to the Relationship Question. Against this, I argue that the questions are independent, and that their answers compatible. I take the two questions to be unrelated unless connected via Maximality, and must be kept separate so as to understand all the rival mereotopological models of pregnancy.

## II. THE TIMING QUESTION

The Timing Question assumes that there is one organism of a certain kind before conception and at least two after birth, and so it asks where between conception and birth (or even after) does the new organism(s) start to exist? Smith and Brogaard’s (2003) paper “Sixteen Days” serves to answer the Timing Question with respect to human beings, and their answer is, as is made clear by the title of their paper, sixteen days into the pregnancy. Therefore, there will be two or more humans from sixteen days—the Gravida and the Foster(s). This type of answer is what I call the “During-Pregnancy” model, where a new organism starts to exist during the pregnancy (which includes at conception). In contrast, Kingma’s (2020) response, “Nine Months” shows that according to the conditions that Smith and Brogaard layout, it is actually at birth when a new human being starts to exist. Therefore, there will only ever be one human during pregnancy—the Gravida—because the Foster does not qualify (even though the baby after birth would). This type of answer is what I call the “After-Pregnancy” model, because it postulates that a new organism starts to exist only after the pregnancy (which includes at birth). I do not wish to challenge these answers to the Timing Question, rather the methodology of how they arrived at those answers.^10^ Specifically, I take issue with how they defend their answers to the Timing Question by providing an answer to the Relationship Question. I take these questions to be separate, and to demonstrate I outline the During-Pregnancy and After-Pregnancy models, and show how they do not entail and are not entailed by any answer to the Relationship Question.

### During-Pregnancy Model

In this model, we count only one human being before the pregnancy, and then at some point during the pregnancy from conception and before birth we count two (or more) human beings. At some time during the pregnancy from conception and before birth, a new human being comes into existence, because the Foster does meet the conditions of being an organism of the relevant kind (which in this case is a human organism) from that point onwards. Therefore, this During-Pregnancy model is committed to there being more than one human being at some point in time during the pregnancy. What this model is *not* committed to is how one human being is related to the other—whether one is *a proper part of* the other, or whether one is *merely contained by* the other. Stating that there is more than one human being in a pregnancy does not, on its own, entail any specific metaphysical relationship between them. It only entails that there is a time during the pregnancy that the Foster has the status of being an organism of the relevant kind.

Smith and Brogaard put forward certain conditions that must be met in order for a thing to qualify as a human being, and a substantial change must occur in the Foster for it to meet those conditions. They argue that “empirical consideration of the biology of pre- and post-gastrular development then allows us to identify the relevant substantial change as occurring at the very end of the sixteen day period” (Smith and Brogaard, 2003, 69). Here they arrive at their answer of sixteen days by creating a list of conditions for being a human being, and seeing where in the pregnancy the Foster meets those conditions. Crucially, one of Smith and Brogaard’s conditions to be a human being is *to be a maximal entity*. What they mean by this is for it to not count as a proper part of anything else, unless it is merely contained within the remainder of that other thing.^11^ Smith and Brogaard plausibly had Maximality (understood in the traditional sense with a capital “M”) in mind, where the term “maximal” is applied to entities *F* that abide by a condition that looks like this:

*Maximality*: Proper parts of an *F* are not themselves *F*s.^12^

Smith and Brogaard make explicit use of maximality in their argument as such:

Maximality will, however, bring the additional advantage that it will throw light on the precise nature of the relationship between [Foster] and [Gravida]. (2003, 69)

However, Maximality can only “throw light” on the relationship between the Foster and Gravida by being incompatible with the conjunction of the Foster being a human and the Foster being a proper part of a human. Maximality thus serves to make “being a human” and “being a proper part of a human” mutually exclusive. If maximality is to make a difference at sixteen days to the existence of a human being, it must be that all other conditions of being a human are met before sixteen days, apart from maximality. However, if the Foster is *never* a proper part of the Gravida, then maximality does not contribute to their argument of why it is at sixteen days that the Foster is a human being and not before. So, for maximality to make a difference to the Timing Question, it must be combined with an answer to the Relationship Question.

As we see later in Section III, Smith and Brogaard *do* provide an answer to the Relationship Question: they hold a *mere-containment* view of the relationship between Foster and Gravida, such that the Foster is not a proper part of the Gravida, but is merely contained by the Gravida. Given that the Foster is never a proper part of the Gravida, their considerations of maximality should not make any difference to their argument regarding how they answer the Timing Question—maximality just does not apply when there is no parthood relation involved. So perhaps instead their considerations of maximality help them in answering the Relationship Question, but again this seems not to be the case, because if the Foster is a maximal entity, then either the Foster is a proper part of the Gravida for as long as that Gravida has another proper part which merely contains the Foster, or the Foster is not a proper part of the Gravida at all. The latter is for what they opt, and the former is dismissed. Now the problem I see here is that they seem to use maximality to underpin an argument for human-hood at sixteen days to non-parthood, *and* they use non-parthood to motivate human-hood at sixteen days. Nonetheless, independent arguments must be provided for why maximality is correct, why “being human” is a maximal property, and why the Foster is not to be considered part of the Gravida from sixteen days on.

### After-Pregnancy Model

The other category of answer to the Timing Question is that a new organism comes into existence at the time of birth or after pregnancy.^13^ During the time throughout pregnancy, there is only one organism, namely the Gravida. The Foster is therefore *not* classed as an organism of the relevant kind. Kingma, though she notes the advantages of this After-Pregnancy model, also critiques it:

It seems so incredibly counterintuitive. It states that the foster does not survive birth, and baby is not the same as foster that existed seconds before. (2018, 177)

I take there to be something wrong with this critique. To say a newborn baby is not “the same” as the Foster is confusing, as on an After-Pregnancy model the Foster is not an organism of the relevant kind anyway. Perhaps, instead, a Foster goes from being one kind of organism to another, the latter being of the relevant same kind as the pre-pregnant Gravida. However, one should not hold that Fosters are organisms during pregnancy just in order to preserve something that persists through birth.

If the Foster is in many ways identical to the newborn baby, then for Smith and Brogaard there is not a substantial enough change in the entity to qualify a difference from Foster to human. They see the transition at birth to be a “mere passage of an entity from one environment to another” (Smith and Brogaard, 2003, 65). It thus seems that they interpret the change from gastrulation to the onset of neurulation to be more substantial than the change at birth. Now, is there *really* a greater change in the Foster during the gradual process of pregnancy, than there is during the abrupt shift at birth? Their view that the change at birth is unsubstantial *relies* on a mere-containment view, where a Foster is contained in the Gravida’s “environment,” and so the change is just the Foster moving from inside to outside a Gravida. This is evident when they immediately clarify the change in environment at birth as being “analogous to an astronaut leaving her spaceship” (Smith and Brogaard, 2003, 65). Now the illegitimate circularity arises, given that they hold a mere-containment view due to the Foster being human at sixteen days.

An example of a more substantial change than change in environment would be when one thing stops being part of another, but this too is circular: it cannot both be the case that (1) at time *t* a human being comes into existence *because* the Foster stops being part of the Gravida and (2) at time *t* the Foster stops being part of the Gravida *because* the Foster becomes a human being. These changes may happen simultaneously, but they cannot be the *reason* for each other. A change in the relationship affects a change in the number of humans (and vice versa) if we have independent grounds for Maximality or the like. Without Maximality, the view that a human being comes into existence at birth would not require that the new human being is not part of the Gravida at the same point in time. Because we normally take the newborn baby *not* to be part of the Gravida, After-Pregnancy models are ordinarily compatible with Maximality.^14^

### Timing and Counting

So far, I have discussed the question of when a new organism of the relevant kind starts to exist. This is related to biological individuality and how we count organisms (as a type of biological individual). In order to say when an organism starts to exist, we need to be able to identify the biological individual that starts to exist. So, when we look at a pregnancy, instead of asking (across time) *when* an organism comes into existence, we can ask (at a time) *how many* organisms there are in existence at that time. Call this the Counting Question, which I take to be a reframing of the issues addressed in the Timing Question. There are many accounts for how we individuate biological entities and thus for how we count those entities, but this is not the place for me to consider such accounts.^15^ Rather, what I want to point out here is that whatever account of biological individuality we take, it helps us to answer the Counting Question (and the Timing Question) but is independent of the Relationship Question unless by explicit connection via a Maximality principle or equivalent.

Say that at a time in the pregnancy, our account of biological individuality counts just one biological individual. As such, we have an answer to the Counting or Timing Question. Is this enough to give us an answer to the Relationship Question, and conclude that the Foster is a proper part of the Gravida? No, it is not. This is because the Foster may not qualify as a biological individual (which would be a thing presumably with bona fide boundaries rather than fiat boundaries meeting certain biological conditions), yet may still be merely contained by the Gravida. In this picture, then, we would have one biological individual (presumably the Gravida), potentially contained in or as a part of that biological individual we have an entity that does not qualify as a biological individual (because certain conditions need to be met for an entity to be a biological individual). The intuitive pull away from this is that if something is contained, then there is a *thing* to be counted. Notwithstanding, one must keep in mind that such a contained thing need not qualify as a biological individual, given the constraints on what it takes to qualify as being such a thing. As such, no answer to the Relationship Question falls out of an answer to the Timing or Counting Question in this way.

Now, say that at another time in the pregnancy our account of biological individuality counts two biological individuals, thus answering our Timing or Counting Question. Is this enough to conclude an answer to the Relationship Question, such that the Gravida merely contains the Foster? No, again, it is not. This is because the Gravida (a biological individual) may have the Foster (another biological individual) as a proper part. Unless we hold that no biological individual can be a proper part of another biological individual, the issues are unrelated. Many argue that biological individuals are made up of biological parts, where those parts also qualify as biological individuals themselves—this is especially prominent in organism-centered views of biological individuality.^16^ Therefore, just as Maximality serves to connect the Timing Question with the Relationship Question, Maximality (or something like it) is required to connect the Counting Question with the Relationship Question, to say whether a biological individual can be a part of another biological individual or not.

Note, however, that being a biological individual is a weaker claim than being an organism, as an organism is a type of biological individual.^17^ So, if the Foster does not qualify as a biological individual, then the Foster would not qualify as an organism. Then what is it? It is biological matter after all, and it seems to be individuated enough to be a unified thing that is considered to be located inside the Gravida. So, why is this not enough to be regarded as a biological individual? Well, it depends on which account of biological individuality you hold, and I do not wish to endorse one here. I accept that it may seem counterintuitive to hold that one thing is part of another or that one thing is contained by another, and still only count one biological individual (despite there being more than one “thing”). The point that I want to make here, though, is that there may be an account of biological individuality that disqualifies the Foster from being a biological individual whilst it being individuated enough to be a thing that is merely contained by the Gravida. Once again then, it seems that without some explicit endorsement (and justification) of a Maximality condition, one cannot learn about the number of human beings from the way that the entities are mereologically related to each other, nor can one learn about the way the entities are mereologically related to each other simply by counting how many human beings there are.^18^

## III. THE RELATIONSHIP QUESTION

Having categorized two answers to the Timing Question, I now embark on outlining many potential answers to the Relationship Question. We arrive at them by answering the following quick-fire questions: (1) Is the Foster a proper part of the Gravida?, (2) Does the Gravida merely contain the Foster?, (3) Do the Gravida and Foster share parts?, and (4) What is the remainder of the entity without the Foster?

### Parthood View

**Figure 1:**
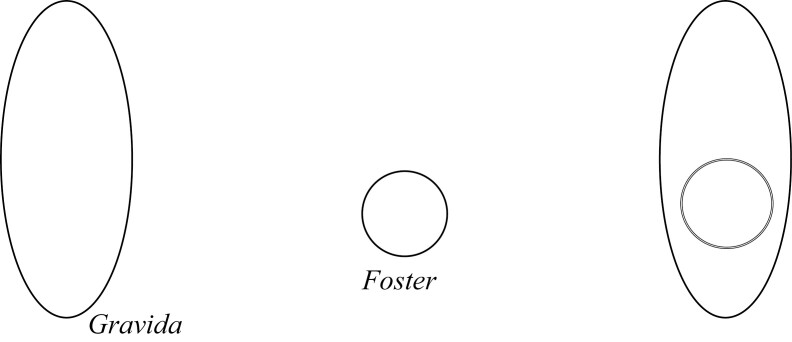
Parthood pregnancy.

This view is put forward by Kingma, who, in rethinking pregnancy, says “one can start by treating talk of fosters being parts of gravidae as parallel to talk of, say, kidneys being parts of dogs” (2019, 612). The Foster and Gravida are not separate, but related as a proper part is to the whole. This can be shown in the following diagram (Fig. 1), where the Gravida is the lined area and the Foster is part of that area circled in black:

Answers to quick-fire questions: (1) *Yes* the Foster is a proper part of the Gravida; (2) *No* the Gravida does not merely contain the Foster; (3) *Yes* they share parts; and (4) The remainder of the entity without the Foster is just the other parts of the Gravida.

I agree with Kingma that if we hold the Parthood view, then we are presented with two options: the Foster is an organism of the relevant kind and so one organism is a proper part of another of the same kind, or the Foster is not an organism of the relevant kind and only becomes so at or after birth.^19^ Kingma states this as a dilemma since she sees both options as being counterintuitive: organisms being a proper part of the same kind of organism, or organisms of the relevant kind (in being of the same kind as the pre-pregnant Gravida) not existing before birth.^20^ In other words, the following three statements (A), (B), and (C), are incompatible, and so only two can be consistently held at one time and the other needs to be rejected:

(A) The Foster is an organism of the relevant kind.
(B) The Foster is a proper part of the Gravida.
(C) No organism can be a proper part of the same kind of organism.^21^

(A) is the During-Pregnancy model, (B) is the Parthood view, and (C) is the Maximality condition. This is Kingma’s dilemma: to reject (A) or reject (C), since she holds (B). But since both are available options, it shows that the mereological position held in (B) does not give us one distinct answer about the number of organisms of the same kind during a pregnancy, and so the Relationship Question does not determine a unique answer to the Timing Question. An answer to the Relationship Question only helps to answer the Timing Question in conjunction with other commitments (like Maximality) that themselves need explicit identification and independent justification. Holding (B) forces one to reject (A) or (C); however, this is only counterintuitive if it really is *intuitive* to hold *both* the During-Pregnancy model *and* Maximality; otherwise, the dilemma is not much of a dilemma—it is more like a choice between two plausible options. Therefore, the Parthood view alone does not dictate what sort of entity the Foster is. If one wants to hold Maximality and the During-Pregnancy model, then one needs to reject (B) by holding some other view of the relationship between Gravida and Foster, like the one presented next.

### Containment View

**Figure 2:**
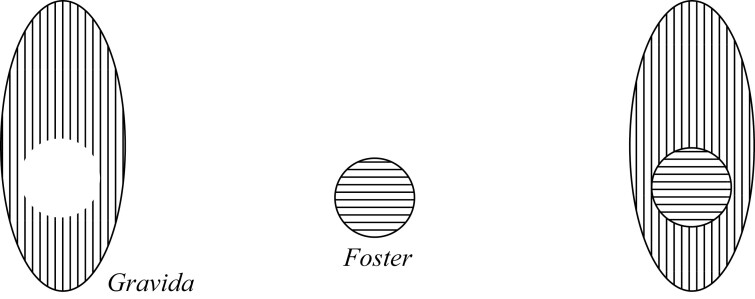
Containment 1 Pregnancy.

According to the Containment view, a Gravida is literally a *mere container* for the Foster. Unlike the Parthood view then, the Gravida and Foster are separate. Smith and Brogaard appear to hold this view, because they understand the Gravida to be a special type of container, namely, having a “niche,” where the Foster is the “tenant.”^22^ On the relation between niche and tenant, they say: “a niche is a part of reality into which an object fits…the niche surrounds its tenant. Moreover, the niche-tenant relation must involve some sort of cavity… in which the tenant is contained” (Smith and Brogaard, 2003, 70). The Foster, as a tenant, inhabits the Gravida, which has a niche (which I call being a niche container).^23^ Smith and Brogaard provide the analogy of the Foster being inside the Gravida in the same way that “a tub of yogurt is inside your refrigerator” (2003, 74). This can be represented in Fig. 2; now the Foster is not part of that vertical lined area but, rather, is the horizontal lined area located inside a niche container within it.

Notice that there are a few variants of this Containment view, where the Foster as the tenant does not completely fill the niche in the Gravida, for example.

**Figure 3:**
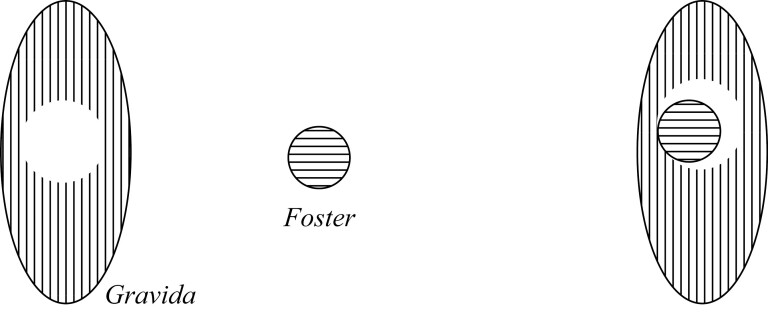
Containment 2 pregnancy.

**Figure 4:**
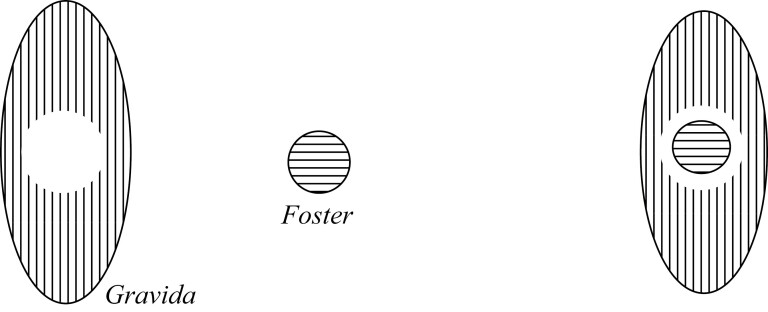
Containment 3 pregnancy.

Figs. 2–4 all give the same answers to the quick-fire questions and thus appear to be mereologically equivalent: (1) *No* the Foster is not a proper part of the Gravida; (2) *Yes* the Gravida does merely contain the Foster; (3) *No* they do not share parts; and (4) The remainder of the entity without the Foster is the Gravida.

The way to demarcate the difference between the Containment views is by utilizing the notion of “contact” in topology.^24^ In Fig. 2, we have a case of complete contact between the Foster and Gravida, in Fig. 3, we have a case of partial contact, and in Fig. 4, we have a case of no contact. Importantly, these Containment views do not state what sort of thing the Foster is; namely, they do not (on their own) dictate whether the Foster is an organism or not—they only say that the Foster is a tenant in a niche container. To do otherwise would again require an explicit commitment to something like a Maximality condition.

### Overlap Views

**Figure 5:**
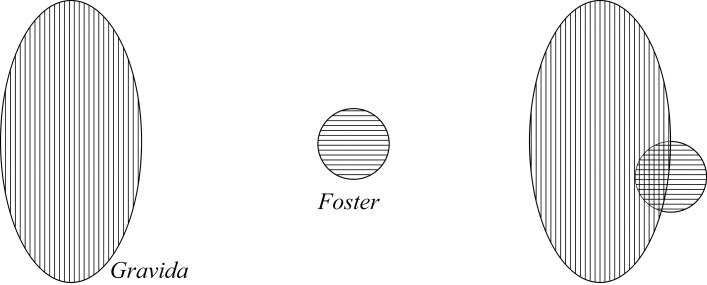
Proper-overlap 1 pregnancy.

Now, we look at the option of the Foster and Gravida sharing parts, or overlapping. When the overlap is between proper parts, we may call it “proper-overlap.” Using this notion we can define models that hold that there are two entities, the Foster and Gravida, which overlap somewhere (but not everywhere, for either entity), and as such share a proper part (rather than an improper part which may include the entirety of one of the entities). This model neither commits to what type of entity the Foster or the Gravida are nor what proper part it is that they share. Therefore, there are a couple of ways that the Foster and Gravida can proper-overlap, resulting in two pictures for this model. I start with the option whereby the proper-overlap is pictured like an overlapping Venn diagram. This can be shown in the diagram (Fig. 5), with the Gravida vertically lined, the Foster horizontally lined, and their shared part lined both vertically and horizontally to create a criss-cross.^25^

**Figure 6:**
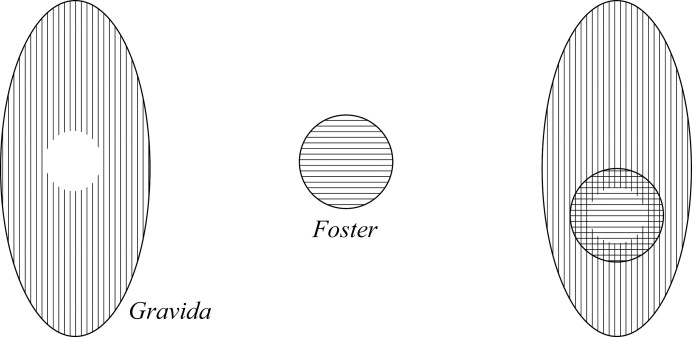
Proper-overlap 2 pregnancy.

In order to visualize the other way of the Foster and Gravida proper-overlapping, picture again the Containment view. Imagine that the Gravida has a niche-like^26^ space for the Foster, but that space is not quite “big enough” for the Foster, so that the Foster and Gravida have a proper-overlap—a shared proper part. This can be represented in the following diagram (Fig. 6), where the same shade coding applies:

Figs. 5 and 6 give the same answers to our quick-fire questions, and so appear mereologically equivalent: (1) *No* the Foster is a not a proper part of the Gravida; (2) *No* the Gravida does not merely contain the Foster; (3) *Yes* they share parts; and (4) The remainder of the entity without the Foster is the Gravida missing its shared parts.^27^

**Figure 7:**
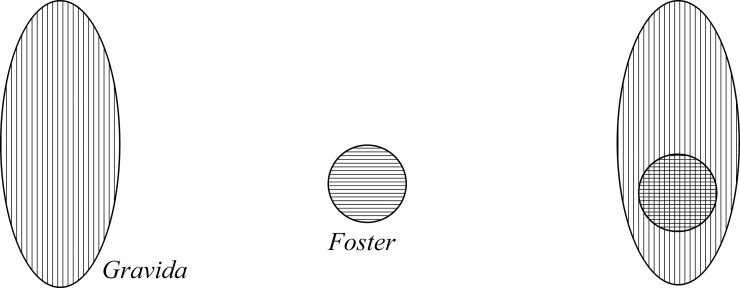
Improper-overlap pregnancy.

Now imagine that the criss-cross area *is* the Foster, such that the shared part between the Gravida and the Foster is a proper part of the Gravida but an improper part of the Foster. This can be pictured again in terms of something like a Venn diagram, but this time where the Foster is completely overlapped by the Gravida, as in Fig. 7.

Figs. 1 and 7 give the same answers to our quick-fire questions, and as such also appear mereologically equivalent: (1) *Yes* the Foster is a proper part of the Gravida; (2) *No* the Gravida does not merely contain the Foster; (3) *Yes* they share parts; and (4) The remainder of the entity without the Foster is just the other parts of the Gravida.

The difficulty with the view represented in Fig. 7 lies in what to call the criss-cross area. All that the criss-cross area as currently shaded represents is that which is both the Foster itself and the Foster as a proper part of the Gravida, but we should be careful not to double-count. Likewise, with the Parthood view represented in Fig. 1, the Foster is a proper part of the Gravida, but is also contained within the external boundary of the Gravida. Even so, we do not count the Foster twice (once as a part and once as being contained). In other words, we do not shade the Foster differently in the Parthood view, because doing so is misleading by assuming that there is more than one entity present. This helps us to see how Improper-Overlap and Parthood are mereologically equivalent. Similarly, given that Figs. 5 and 6 are mereologically equivalent, I do not count them as distinct options for ways of modeling the relationship between the Foster and Gravida. Topological tools would be able to differentiate them, since it is only in topological terms of location and contact that they differ. For now though, I conflate the cases of proper-overlap as ways of picturing the same view, which I simply call the Overlap view from hereon.

### Underlap View

**Figure 8:**
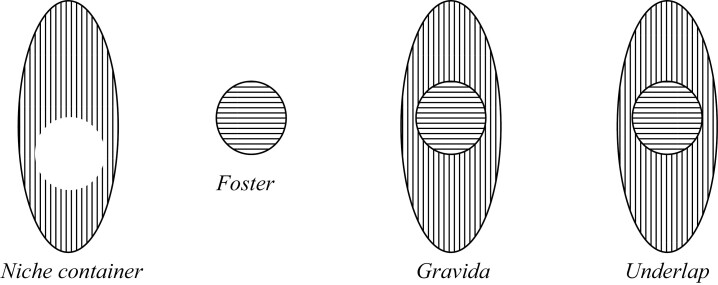
Niche-container pregnancy.

The final view I briefly describe is the Underlap view, according to which the Gravida is the minimal underlap (or mereological sum) of the niche container and the tenant (Foster), where the niche container is identified as the maximal remainder of the Gravida minus Foster. This Underlap view can be represented in Fig. 8, with a niche container and a Foster, which together compose to form the Gravida.^28^

Answers to quick-fire questions: (1) *Yes* the Foster is a proper part of the Gravida; (2) *No* the Gravida does not merely contain the Foster (but the remainder of the Gravida minus Foster does merely contain the Foster); (3) *Yes* they share parts; and (4) The remainder of the entity without the Foster is a niche container, not the Gravida.

There is an important distinction between the Containment view and the Underlap view, and this has to do with the entities they identify. The Containment view treats the Gravida as being a niche container for the Foster. When we take the mereological sum of the Gravida and the Foster, we are left with an entity (the underlap) that is not specified as a particular thing itself. The Containment view identifies the underlap as some arbitrary mereological sum rather than the Gravida, whereas the Underlap view identifies the underlap as the Gravida itself. When the Gravida *is* the underlap, the Gravida will have a proper part that is the Foster, *and* a proper part that is a niche container for the Foster. This makes the Underlap view a hybrid whereby there is both mere-containment and proper parthood.^29^ So, the Foster being a tenant in a niche does not alone establish that the Foster is not *also* a proper part of the Gravida, as the Gravida may not be identified with the niche container but with the underlap of the niche container and tenant, thus allowing for the Foster to be a proper part of and contained within the Gravida.

There is also an interesting and important difference between the Underlap view and Parthood view, which again is due to the entities the views identify and whether they are demarcated by fiat or bona fide boundaries. Specifically, the difference between the views is due to the identification of the Gravida minus the Foster. On the Parthood view, when we remove the Foster part from the Gravida whole, we are not left with a niche container for the Foster, rather we are left with a Gravida missing one of its parts: this remainder on the Parthood view is identified no further, other than being a gerrymandered remainder, and as such there is only a fiat boundary between that remainder and the Foster. Whereas, on the Underlap view, when we remove the Foster from the Gravida, we are left with a niche container for the Foster: this remainder on the Underlap view is thus identified and named in an ontologically privileged way, and as such there is a bona fide boundary between the niche container and the Foster. This is because “niche container” is stronger than “remainder” in that there are more conditions on what it takes to be a niche container than what it is just to be the remainder, such that the former is demarcated with bona fide boundaries and the latter only with fiat boundaries. In the Underlap view, the Gravida has both a niche container and a tenant as proper parts, whereas on the Parthood view the Foster is a proper part of the Gravida, but there is no other distinguishable proper part which counts as a niche container for the Foster.

I have now finished articulating four distinct views: Parthood, Containment, Overlap, and Underlap. Kingma aims to argue for the Parthood view; however, it may be that her arguments do not point in favor solely of that view. It is worth mentioning that any argument against the Underlap view cannot appeal to a Maximality principle if it is to work in favor of the Parthood view. It might be that what is objectionable about the Underlap view is how many entities it identifies (a Foster, a Gravida, *and* a niche container), where the niche container and Gravida may be of the same kind. Maximality would rule this possibility out. Now, if entities being part of entities of the same kind in the Underlap view is unacceptable, then similar reasons apply against the Parthood view too, where we may similarly see a Foster being a part of a Gravida of the same kind. As such, one cannot reject Maximality in favor of Parthood and simultaneously endorse Maximality against Underlap.

**Table 1.** Combinations

Model	(A) During-Pregnancy	(B) After-Pregnancy
**(1) Parthood**	(i) Yes: F is a proper part of G (ii) No: F not merely contained by G (iii) Yes: F and G do share parts (iv) Remainder: G missing a part (v) Yes: F is organism of rel. kind Incompatible with Maximality	(i) Yes: F is a proper part of G (ii) No: F not merely contained by G (iii) Yes: F and G do share parts (iv) Remainder: G missing a part (v) No: F is not organism of rel. kind Compatible with Maximality
**(2) Containment**	(i) No: F is not a proper part of G (ii) Yes: F merely contained by G (iii) No: F and G do not share parts (iv) Remainder: the whole of G (v) Yes: F is organism of rel. kind Compatible with Maximality	(i) No: F is not a proper part of G (ii) Yes: F merely contained by G (iii) No: F and G do not share parts (iv) Remainder: the whole of G (v) No: F is not organism of rel. kind Compatible with Maximality
**(3) Overlap**	(i) No: F is not a proper part of G (ii) No: F not merely contained by G (iii) Yes: F and G do share parts (iv) Remainder: G missing a part (v) Yes: F is organism of rel. kind Compatible with Maximality	(i) No: F is not a proper part of G (ii) No: F not merely contained by G (iii) Yes: F and G do share parts (iv) Remainder: G missing a part (v) No: F is not organism of rel. kind Compatible with Maximality
**(4) Underlap**	(i) Yes: F is a proper part of G (ii) No: F not merely contained by G But F is merely contained (iv) (iii) Yes: F and G do share parts (iv) Remainder: niche container for F (v) Yes: F is organism of rel. kind Incompatible with Maximality thrice: F, G, and (iv) same kind of organism	(i) Yes: F is a proper part of G (ii) No: F not merely contained by G But F is merely contained by (iv) (iii) Yes: F and G do share parts (iv) Remainder: niche container for F (v) No: F is not organism of rel. kind Incompatible with Maximality twice: G and (iv) are same kind of organism

## IV. COMBINING THE MODELS

I started this paper with the following two metaphysical questions about pregnancy: (*Timing Question*) When does an organism of the relevant kind start to exist? (*Relationship Question*) Is the Foster a proper part of, or merely contained by, the Gravida? I then went on to articulate the following possible answers to these questions: (*Timing Answers*) as described in section II During-Pregnancy model and After-Pregnancy model; (*Relationship Answers*) as described in section III Parthood view, Containment view, Overlap view, and Underlap view. I have not argued for which I take to be the best model of pregnancy, but have instead just aimed to showcase some of the options. I also have not explored the possibility of different models being held at different times throughout the pregnancy. For example, it may be that in the first trimester a certain relationship between the Foster and Gravida holds, and then in the second trimester a different relationship between the Foster and Gravida holds. As such, it may be that for each moment of the pregnancy, we could ask “At *this* time, what is the mereotopological relationship between the Foster and Gravida, and at *this* time, is the Foster an organism of the relevant kind?” This may result in different answers for different time-slices of the pregnancy. For now, we can add a question (5) to our quick-fire questions to delineate the positions at different times: (*Quick-fire questions*) (1) Is the Foster a proper part of the Gravida?, (2) Does the Gravida merely contain the Foster?, (3) Do the Gravida and Foster share parts?, (4) What is the remainder of the entity without the Foster?, and (5) Is the Foster an organism of the relevant kind?

By looking at the answers to these questions, we were able to see which models were mereologically equivalent and which could be further delineated via topology. We found that there are many more options to articulate the metaphysical relationship between the Foster and Gravida than Kingma and Smith and Brogaard describe. Table 1 below shows the ways in which models that outline when a new organism starts to exist can be combined with the views that outline the relationship between the Foster and Gravida. In each combination, I note whether it is compatible or incompatible with a Maximality principle so as to make clear what role such a principle has.

## V. LESSONS LEARNED

In this paper, I set out on an exploration of different mereotopological models of pregnancy, and along the way, I have argued against aspects of the debate found primarily from the exchange in the *Journal of Medicine and Philosophy* between Kingma and Smith and Brogaard that I see as being misguided or incorrect. In this final section of my paper, I bring together those critiques and suggest ideas for future research in this currently minimal but exciting and developing area of philosophy.

Throughout this paper, I have assumed that when we ask whether the Foster is a part of the Gravida, the sort of parthood that we mean is captured by mereology. However, not everyone is on board with this assumption, and some take biological parthood to be not captured by mereology.^30^ If they are right, then given that the Foster and Gravida are biological entities, perhaps we should look to the conditions on biological (rather than mereological) parthood to see if the Foster and Gravida meet such conditions. Whether the Relationship Question should be cashed out in mereological or biological parthood terms has made little difference to me here, but may have interesting consequences for those like Kingma as well as Smith and Brogaard who claim to be doing biologically informed work in metaphysics and ontology. Whether the Relationship Question can be answered by appeal to mereology in metaphysics or embryology in biology has also made little difference to me here, because I have not set out to (or to say how to) adjudicate between these options.^31^

I have looked not only at parthood, but also at containment. Given that parthood is a mereological notion and containment is a topological notion, the Relationship Question as it is posed by Kingma is hard to parse. This is because the two options in the question are from different domains—mereology and topology—and as such are not easily seen as contrast classes.^32^ In order to make the contrast between parthood and containment, I attempted to define *mere-*containment in a way that is incompatible with parthood. Then we saw that there was more than one way for a thing to be contained, and therefore different potential topological relationships between the Foster and Gravida. Some models may be mereologically, but not topologically, equivalent and vice versa. This shows that topology has a place in describing the metaphysics of pregnancy, because without it the mereology is insufficient in capturing all the distinct models that I have laid out here. As such, a fuller picture of the metaphysics of pregnancy requires *mereotopology,*^33^ but I do not take it to capture the *fullest* picture. There are other relations between the Foster and Gravida that are not mereological or topological, for example, *dependence*, which provides a third coordinate for investigating the different models of pregnancy and thus far has not received the same level of attention that Kingma as well as Smith and Brogaard dedicate to the mereological and topological relation between Foster and Gravida. The asymmetrical level of dependence of the Foster on the Gravida for survival may in itself provide evidence for or against a parthood relationship between them; however, the notion of dependence requires much further elaboration for this.

I focused on the two coordinates of parthood and containment in the Relationship Question and how they relate to the Timing Question. I argued that an answer to one cannot help answer the other, unless explicitly connected by extra commitments like Maximality. However, it is worth noting that having a mereological or topological relationship between things requires or at least implies that there are multiple things to relate. So, it seems like the mere appropriateness of asking a Relationship Question answers a certain Counting Question as to how many *things* there are, since we can identify and relate more than one. Now my point is that this still does not say what *kind* of things they are, or when things become a member of a certain kind. We may be able to individuate distinct things as being parts of or contained by another, but this does not answer whether these things are organisms or biological individuals. Hence, this leaves the Timing Question unanswered by the Relationship Question. Furthermore, one should not circularly argue for an answer to the Timing Question by using an answer to the Relationship Question whilst also motivating an answer to the Relationship Question by appealing to an answer to the Timing Question. I took Smith and Brogaard to be arguing this way and objected to their paper on that basis.

I also took issue with the way Maximality has been presented in the debate thus far. I have argued that Maximality serves to make “being a human” and “being a proper part of a human” mutually exclusive. Kingma takes this to force a dilemma upon those who hold a Parthood view, where they must either reject Maximality or the During-Pregnancy model. Here, this to me seems not to be a dilemma instead just a choice, as it has not yet been argued that both Maximality and During-Pregnancy are intuitive to hold at the expense of a Parthood view. I also objected to the way that Smith and Brogaard invoke maximality in their own sense to be a condition on being a human (or more generally, a substance). The problem was that they seem to use maximality to argue *both* from human-hood at sixteen days (an answer to the Timing Question) to non-part-hood (an answer to the Relationship Question), *and* from non-part-hood to human-hood at sixteen days. To use maximality in one direction without justification is bad enough, but to use it in both directions is problematically circular. If Maximality is to make a difference to one’s views on pregnancy, it needs independent justification that is stronger than any counterexample to Maximality (that may include pregnancy itself!). This leads into meta-philosophical methodological territory that I discuss elsewhere.^34^

As for why all of this matters, given that Kingma poses the question as: “Were you a part of your mother?” in her *Mind* article, one of the implications may be that we learn what we once were—namely, a part of our mothers or not. This has intrinsic value, simply because truth is valuable.^35^ Nonetheless, it also has instrumental value, in that it may be that there are ethical consequences of such metaphysical views (though these are not straightforward). For example, Smith and Brogaard connect their position on when a human starts to exist with ethical issues in abortion and stem cell research:

What follows is an exercise in ontology, and clearly no conclusions of an ethical sort can be drawn directly from the answer to any ontological question… It seems to us, however, to be equally clear that an answer to the question as to when a human organism begins to exist can be of some help in settling the difficult problems which arise in connection with the issue of abortion and embryonic stem cell experimentation. (2003, 46)

I agree that, given the complexity of the debates in reproductive ethics, the metaphysical models I have outlined are *not* on their own (as “exercises in ontology”) sufficient to determine the legitimacy or illegitimacy of any particular problematic case, because the models alone do not determine, for example, the rights that a human Gravida has over her body or the produce of her body, nor the moral status of the Foster or what sort of thing the Foster is. However, many of the reasons that we cite to support our stance on reproductive issues are based (sometimes unknowingly) on metaphysical grounds (though admittedly the grounds are not so much regarding the relationship between Foster and Gravida but more commonly regarding what sort of thing the Foster is). As such, it is important to look carefully at these metaphysical grounds if they are to be used as motivation for or against a bioethical issue and challenge any metaphysical assumptions that may have been made. Kingma notes:

The parthood view may also prompt a reconsideration of various ethical and legal questions, particularly where work in these domains has presupposed a containment view of pregnancy. For example, those who discuss or invoke certain moral and legal rights and principles… typically assume that the parties involved have separate bodies. So how, if at all, are these to be understood when it comes to a situation in which one body is a part of another? This is an open question, but one which must, in light of the parthood view, be settled by any who want to invoke such rights or principles in ethical and legal debates that concern pregnancy. (2019, 640)

Such domains that seem to have presupposed a Containment view of pregnancy include surrogacy and ectogenesis. I (and others) have discussed these elsewhere.^36^ There are other areas of reproductive bioethics where the metaphysics of pregnancy can have an impact, and I leave that for future work and for the readers to ponder. For now, it suffices to say that the metaphysics of pregnancy matters.

## VI. CONCLUSION

To summarize, in this paper, I have shown that there is more than one way to think about the metaphysics of pregnancy, and more than one question to ask about it. I have argued that these questions are distinct, and that the full sets of combinations of their answers are compatible. The way to connect the questions regarding when a new organism of the relevant kind starts to exist with how a Foster is related to the Gravida is via a Maximality condition that prevents one organism being a proper part of another organism of the same kind. But, such a Maximality condition need not be held, and it is unclear whether Maximality constrains what we say about pregnancy, or whether pregnancy constrains what we say about Maximality. In general, in this paper, I have helped to lay the foundation for future work in the metaphysics of pregnancy by distinguishing and clarifying the questions that need to be answered, by showing the need to independently justify Maximality, and by mapping out some of the (mereotopo)logical space of the various models of pregnancy that we may hold.

## Footnotes

1 See Smith and Brogaard (2003) and Kingma (2020) both in *the Journal of Medicine and Philosophy*. See also Kingma (2019) and Kingma (2018).

2 See Smith and Brogaard (2003, 45) and Kingma (2020).

3 See Alvargonzalez (2016), Damschen et al. (2006), Koch-Hershenov (2006), Lee, Tollefsen and George (2014), Morris (2012), and Smith and Brogaard (2003, 46). This list is from Kingma (2020).

4 Making use of “kinds” is problematic; yet, it is hard to avoid, given my use of Maximality in this paper.

5 This is the way the question about the metaphysical relationship is posed by Kingma (2019).

6 Thanks to an anonymous reviewer for another example of uncontained parts: the extended mind thesis (Clark and Chalmers, 1998). According to that thesis, a part of one’s cognition is not contained within one’s body or brain, but rather is outside of it. While outside of it, it is still a functioning part of the whole. Whereas, in the shelves outside of the oven case, the shelves are no longer serving the function of being a part of the oven. I use the example of the oven since pregnancy is often colloquially described as a case of “buns in the oven,” which I go on to call the Containment view.

7 See Simons (1987) for a detailed account of the parthood relation and definition of proper parthood.

8 See Smith and Varzi (2000) for a discussion of the distinction between fiat and bona fide boundaries.

9 By “merely,” I do not mean that the Gravida plays no other role. Rather, I use the term “mere” to draw attention to the notion of “containment” in what I call the Containment view ruling out a parthood relationship (since on what I call the Parthood view there is still some containment as the Foster is inside the Gravida, but it is not “mere” containment, since there exists a parthood relationship there too). “Mere,” therefore, only serves to rule out parthood.

10 See also Kingma (2020) for a critique of Smith and Brogaard (2003).

11 This is their exact wording: “An entity x is maximal if and only if every entity y which (1) satisfies conditions 1–11 [for being an organism] and (2) has x as proper part, has some other proper part which is a niche for x” (Smith and Brogaard 2003, 71). It follows from this that an organism x cannot be a proper part of another organism y unless y has a proper part that contains a niche for x. (Niche will be defined later.)

12
Sider (2001, 1). Sider specifically says “large parts of an *F* are not themselves *F*s.”

13 I have not given an account of what “birth” is, but I use the intuitive understanding of birth as the end of labor (after “*l*”) when Foster has emerged “outside” the Gravida and the umbilical cord is cut.

14 I do not discuss the possibility of the newborn baby (the once-was-Foster) being a proper part of the mother (the once-was-Gravida), nor the possibility of the newborn baby being contained by the mother. I take them to be counterintuitive, and I only concentrate on the relationship *during* pregnancy.

15 See Grose (2020) for how different accounts of biological individuality give different answers to a Counting Question. Wilson and Barker (2018) give an overview of biological individuality.

16 See Jagers op Akkerhuis (2010) and Pepper and Herron (2008) for such organism-centered accounts.

17 Thanks to an anonymous reviewer for noting that one definition of “organism” in particular sees it as a living self-organizing whole with its own biological interests, making it hard to imagine how such a whole could also be part of another whole whose biological interests appear sometimes to diverge. But, not all biological entities have “interests,” kidneys, for example, and as such what qualifies as an organism in this sense must also be the right sort of thing to be said to have its own interests (and presumably Fosters and Gravidas do indeed qualify as entities that have interests). The issue of conflicting interests between the Gravida and Foster is often called the “maternal-fetal conflict”—see Haig (1993)—yet this need not entail that one cannot be in conflict with the other as a part of itself.

18 Thanks to an anonymous reviewer for pointing out that if we are to count two human beings, A and B, we can know at least one polyadic property about them, namely, that A ≠ B. If identity is defined relationally, and we have two different things, then we can know at least one relational property about them: that they are not numerically identical. But, the relation of non-identity is not the relation in question, rather the relation of being part of another is of interest—a mereological relation.

19
Kingma (2018). Note that the way Kingma presents this confuses the debate slightly between an organism’s not existing during pregnancy and it is not being of the right kind during pregnancy.

20 Here, I assume that after birth the Foster is not a part of the Gravida, whether it was before or not.

21 Notice that (A)–(C) is general, whereas Smith and Brogaard’s argument turns on what it is like to be a *human being* specifically. There may be further questions as to whether a Foster can be an organism of a *different* kind to the Gravida, and whether Maximality extends to organisms being part of *any* organism.

22 Not all cases of mere containment involve a niche, because some mere containers do not meet the further constraints to have a niche, but all niche containers are mere containers. This model can be articulated without the extra condition for having a niche, so Smith and Brogaard just hold a *version* of this model.

23 The niche itself is the hole, so the thing *with* the hole is the *niche container*, which contains the tenant.

24 See Casati and Varzi (1997) for a discussion of how to formally represent the contact relation.

25 Perhaps this way of picturing proper overlap should just be rejected because the Foster is not generally contained by the Gravida—it “sticks out” so is not even within the Gravida’s external boundary.

26 Smith and Varzi’s account of what a niche is does not allow it to share parts with tenants (1999, 213).

27 Thanks to two anonymous reviewers for noting examples of where living wholes share some parts with another living whole, as in the case of conjoined twins, or when one can wholly or partially contain (part of) the other whole, as in the case of breastfeeding or coitus. There may also be a case of this during birth, with the emergence from the womb where the Foster does indeed “stick out.” The placenta (and perhaps umbilical cord) could be considered either as a shared part of the Foster and Gravida or as a part of the Foster whilst being outside the external boundary of the Gravida, thereby constituting the criss-crossed shaded area that indicates where the overlap of shared parts is.

28 I am ignoring the cases when the Foster and Gravida also overlap for simplicity. Note that if the Foster and the Gravida do overlap, then the Gravida cannot be considered a niche container.

29 This Underlap view looks similar to a view gestured at in Kingma (2019), footnote 3.

30 See Kaiser (2018), for example.

31 Some believe that the Timing Question can be answered from within the sciences. See, for example, Condic (2009). But, this at least assumes that what the sciences mean by “the start of life” is the same as what it means in philosophy, which it may not be, and further presupposes that the organism whose life span is in question maps on neatly with the entity that philosophers are interested in.

32 Similarly, Casati and Varzi (1997) ask: “How should we deal with the interplay between truly spatial concepts—such as ‘contained in’ or ‘located between’—and purely mereological (part-whole) notions?”

33 This is in line with Varzi (1994) who argues that topology is needed more fully describe reality.

34 See Finn (2021). Other methodological concerns include *how* to find out if the Foster is a part of the Gravida or not: do we (1) take a top-down decomposition approach, and look to the whole Gravida to see if it has a Foster as a part or (2) take a bottom-up composition approach, and look to the Foster and other potential parts to see if they make up a Gravida? Both approaches seem to be problematic. It may be that our intuitions sway us for or against certain mereotopological options, but how these intuitions should be weighed up against our theories (and whether we revise our theories or our intuitions) leads further into metaphilosophical territory that I do not have space to address here.

35 This, in turn, may have implications for the personal identity debate. If we were once fetuses (a claim that depends on how one answers the Timing or Counting Question), then whatever model in the metaphysics turns out to be true about the relationship between fetus and Gravida tells us if we, as fetuses, were part of our gestational mothers or not. This thereby tells us something about our personal identity at the start of our “lives,” as fetuses. For a good overview of the personal identity debate, see the first chapter of Olsen (2007) and Olsen’s (2019) Stanford Encyclopedia entry.

36 See Finn (2018), Finn and Isaac (2021), Kingma and Finn (2020), and Baron (2018).
